# Association of a Healthy Lifestyle With All-Cause and Cause-Specific Mortality Among Individuals With Probable Sarcopenia: Population-Based Cohort Study

**DOI:** 10.2196/65374

**Published:** 2025-07-28

**Authors:** Ning Wang, Yuqing Zhang, Junqing Xie, Na Lu, Aojie Zheng, Changjun Li, Jie Wei, Chao Zeng, Guanghua Lei, Yilun Wang

**Affiliations:** 1 Department of Orthopaedics, Xiangya Hospital, Central South University, 87 Xiangya Road, Kaifu District, Changsha, 410008, China, 86 073184327326; 2 Key Laboratory of Aging-related Bone and Joint Diseases Prevention and Treatment, Ministry of Education, Xiangya Hospital, Central South University, Changsha, China; 3 Hunan Key Laboratory of Joint Degeneration and Injury, Xiangya Hospital, Central South University, Changsha, China; 4 Division of Rheumatology, Allergy, and Immunology, Department of Medicine, Massachusetts General Hospital, Harvard Medical School, Boston, MA, United States; 5 The Mongan Institute, Massachusetts General Hospital, Harvard Medical School, Boston, MA, United States; 6 Nuffield Department of Orthopaedics, Rheumatology and Musculoskeletal Sciences, University of Oxford, Oxford, United Kingdom; 7 Arthritis Research Canada, Richmond, BC, Canada; 8 National Clinical Research Center for Geriatric Disorders, Xiangya Hospital, Central South University, Changsha, China; 9 Department of Endocrinology, Endocrinology Research Center, Xiangya Hospital of Central South University, Changsha, China; 10 Department of Epidemiology and Health Statistics, Xiangya School of Public Health, Central South University, Changsha, China

**Keywords:** healthy lifestyle, sarcopenia, mortality, cohort study, muscle strength

## Abstract

**Background:**

Individuals with probable sarcopenia have shown excess mortality, yet no specific treatment regimen has been established. While lifestyle factors improve health and longevity in general populations, their role in probable patients with sarcopenia remains unclear due to differing lifestyle patterns. Clarifying this could inform strategies to address this unmet need.

**Objective:**

We aim to quantify the impact of a healthy lifestyle on all-cause and cause-specific mortality in probable sarcopenic populations using a large-scale prospective cohort study.

**Methods:**

Participants were selected from the UK Biobank, aged 40‐69 years, during 2006‐2010. Probable sarcopenia was identified according to EWGSOP2 (European Working Group on Sarcopenia in Older People 2) criteria, resulting in 20,654 participants being included in this study. Death dates and underlying causes were obtained from the National Health Service Information Center. Cox proportional hazard models and population-attributable risk were used to assess the associations between healthy lifestyle factors and premature mortality risk.

**Results:**

A total of 20,654 individuals with probable sarcopenia were included in this study. The median age of the population was 62.0 (IQR 56.0-66.0) years, and 60.6% (n=12,528) were women. During a median follow-up duration of 11.5 (IQR 10.8-12.3) years, 2447 participants died. All healthy lifestyle factors, including nonsmoking (*P*<.001), moderate alcohol intake (*P*<.001), regular physical activity (*P*<.001), a healthy diet (*P*=.01), limited television-watching time (*P*<.001), adequate sleep duration (*P*=.001), and strong social connections (*P*<.001), were independently associated with lower mortality risk. To evaluate the cumulative associations between modifiable lifestyle factors and mortality outcomes (all-cause and cause-specific) among patients with probable sarcopenia, we developed a healthy lifestyle index. Participants were assigned one point per adherence to each optimal lifestyle factor. Compared with individuals with 0‐2 healthy lifestyle scores, hazard ratios of all-cause mortality for those with 3 to 6‐7 factors were 0.67 (95% CI 0.59‐0.76), 0.51 (95% CI 0.45‐0.57), 0.43 (95% CI 0.38‐0.49), and 0.33 (95% CI 0.29‐0.39), respectively (*P* for trend <.001). There was also a dose-response relationship between the number of healthy lifestyle factors and mortality from cancer, cardiovascular disease, respiratory disease, digestive disease, and other causes (all *P* for trend<.001). Population-attributable risk analysis indicated that 25.7% (95% CI 22%‐29%) of deaths were attributable to a poor lifestyle (scoring 0‐5).

**Conclusions:**

A healthy lifestyle is associated with a lower risk of all-cause mortality and mortality due to cancer, cardiovascular disease, respiratory disease, and digestive disease among individuals with probable sarcopenia. Adopting a healthy lifestyle (scoring 6‐7) could prevent 25.7% of deaths in this population.

## Introduction

Sarcopenia, a progressive and generalized skeletal muscle disorder, affects approximately 10%‐27% of people older than 60 years of age worldwide [1]. The sarcopenia diagnosis follows a three-stage hierarchy per the 2019 EWGSOP2 (European Working Group on Sarcopenia in Older People 2) guidelines: probable sarcopenia is defined by low muscle strength (primary criterion, measured via handgrip dynamometry); confirmed sarcopenia requires concurrent low muscle mass; and severe sarcopenia is diagnosed with additional functional limitation [2]. Even patients in the mildest stage, probable sarcopenia, exhibit a nearly 1.6 times higher risk of mortality compared to the general population [3-6]. Given the high prevalence and significant adverse consequences of sarcopenia, it is crucial to identify potentially modifiable factors to lower the premature mortality risk among this population.

The EWGSOP2 guideline underscores the critical role of lifestyle management in sarcopenia [7], given that physical activity and dietary interventions have been shown to retard sarcopenia progression and reduce mortality risk in this population [8]. However, current evidence is limited to a narrow range of lifestyle factors, notably neglecting other critical determinants, especially emerging ones such as sleep quality, sedentary behavior, and social engagement [9-11]. These lifestyle factors have already been proven to promote health in the general population by reducing inflammation and comorbid burdens [12-14], which are also relevant to sarcopenia [15]. However, whether and to what extent individuals with sarcopenia can benefit from such multidimensional lifestyle factors remains unclear due to their distinct behavioral patterns; for example, individuals with sarcopenia usually reduce their regular physical activity and tend to be more socially isolated [16,17]. Additionally, there is still a lack of evidence on the combined effects of lifestyle factors among individuals with sarcopenia, despite their strong interrelation [18]. Addressing this evidence gap is critical to inform evidence-based guidelines and population-level strategies for behavior change.

To bridge the knowledge gap, we analyzed data from the UK Biobank study to examine the relation of 7 healthy lifestyle factors, that is, no current smoking, moderate alcohol consumption, healthy diet pattern, regular physical activity, adequate sleep duration, short television watching time, and appropriate social connection to all-cause mortality. We also estimated the proportion of deaths that can be prevented theoretically by simultaneously adopting several healthy lifestyle factors among individuals with sarcopenia.

## Methods

### Study Population

The UK Biobank study is a population-based cohort that recruited more than 500,000 participants aged 40‐69 years during 2006‐2010 [19]. Participants provided detailed health-related data and lifestyle information, had physical measurements taken, and provided biological samples at 22 assessment centers across England, Scotland, and Wales [20].

Handgrip strength was measured using a Jamar J00105 hydraulic dynamometer by a trained nurse in a standardized clinical setting [3]. Participants were seated upright with forearms supported on armrests, and bilateral measurements were obtained via a single 3-second maximal voluntary contraction of each hand. The average of right- and left-hand measurements, expressed in kilograms, was used in this study. Sarcopenia was diagnosed according to the EWGSOP2 criteria [2]. Due to the scarcity of confirmed sarcopenia cases in the UK Biobank [21], participants with probable sarcopenia, defined as handgrip strength <27 kg among men and <16 kg among women, were finally included in this study.

### Ethical Considerations

All participants provided written informed consent before the data collection. The study was approved by the National Information Governance Board for Health and Social Care and the National Health Service North West Multicenter Research Ethics Committee (16/NW/0274; ethics approval for UK Biobank studies). This study was conducted with permission (UKB application 77,646) from the UK Biobank. The UK Biobank has made necessary efforts to safeguard participant privacy and provided appropriate compensation to the participanting subjects; all patients’data have been anonymized and deidentified.

### Assessment of Lifestyle Factors

The impact of seven modifiable healthy lifestyles was evaluated in the current analysis. These included four well-established factors (ie, smoking status, alcohol intake, physical activity, and diet) [22] and three emerging factors (television watching time, sleep duration, and social connection) [12,18,23]. All information on lifestyle factors was self-reported and assessed using a touchscreen questionnaire at baseline. Detailed definitions of each lifestyle factor are shown in Multimedia Appendix 1.

Each lifestyle variable was coded 0 or 1, with 1 indicating the adoption of a particularly healthy lifestyle factor based on the following criteria: no current smoking (never or previous smoker), moderate alcohol consumption (women ≤1 drink per day, and men ≤2 drinks per day regularly), healthy diet pattern (≥5 recommended food component groups), regular physical activity (≥150 min per week of moderate activity, or ≥75 min per week of vigorous activity), adequate sleep duration (7‐8 h per day) [24], short television watching time (<4 h per day) [25], and appropriate social connection (ie, moderately active and active social connection status) [26]. An overall healthy lifestyle score was constructed as the sum of the scores of 7 lifestyle factors, with a higher score indicating higher adoption of an overall healthy lifestyle. For the avoidance of extreme groups with limited cases, we collapsed scores of 0, 1, and 2 into one category and 6 and 7 into another category. The categorization choices are arbitrary but justifiable to avoid floor and ceiling effects.

### Ascertainment of Outcomes

The outcome variables consisted of all-cause mortality and cause-specific mortality, that is, death from cancer, cardiovascular disease (CVD), respiratory disease, neurodegenerative disease, digestive disease, and other causes. Information about the death date and underlying cause was obtained from the National Health Service Information Center (for England and Wales) and the National Health Service Central Register Scotland (for Scotland). The cause of death was defined based on the International Classification of Diseases, 10th Revision code. Detailed information about the linkage procedure is web-accessible [27].

### Assessment of Covariates

Covariates included age (years); sex (women or men); ethnicity (White, Black, Asian, Mixed or other); education (higher, ie, college or university degree or other professional qualification; upper secondary, ie, second or final stage of secondary education; lower secondary, ie, first stage of secondary education; vocational, ie, work-related practical qualifications; or other); social deprivation (Townsend deprivation index); and employment (currently employed or not).

### Statistical Analysis

Baseline characteristics are presented as the mean (SD) if normally distributed or median (IQR) if nonnormally distributed for continuous variables and frequency (%) for categorical variables. The distribution of quantitative variables was evaluated using both graphical assessments (ie, histograms and Q-Q plots) and formal normality tests (ie, skewness and kurtosis metrics).

**Figure 1.**
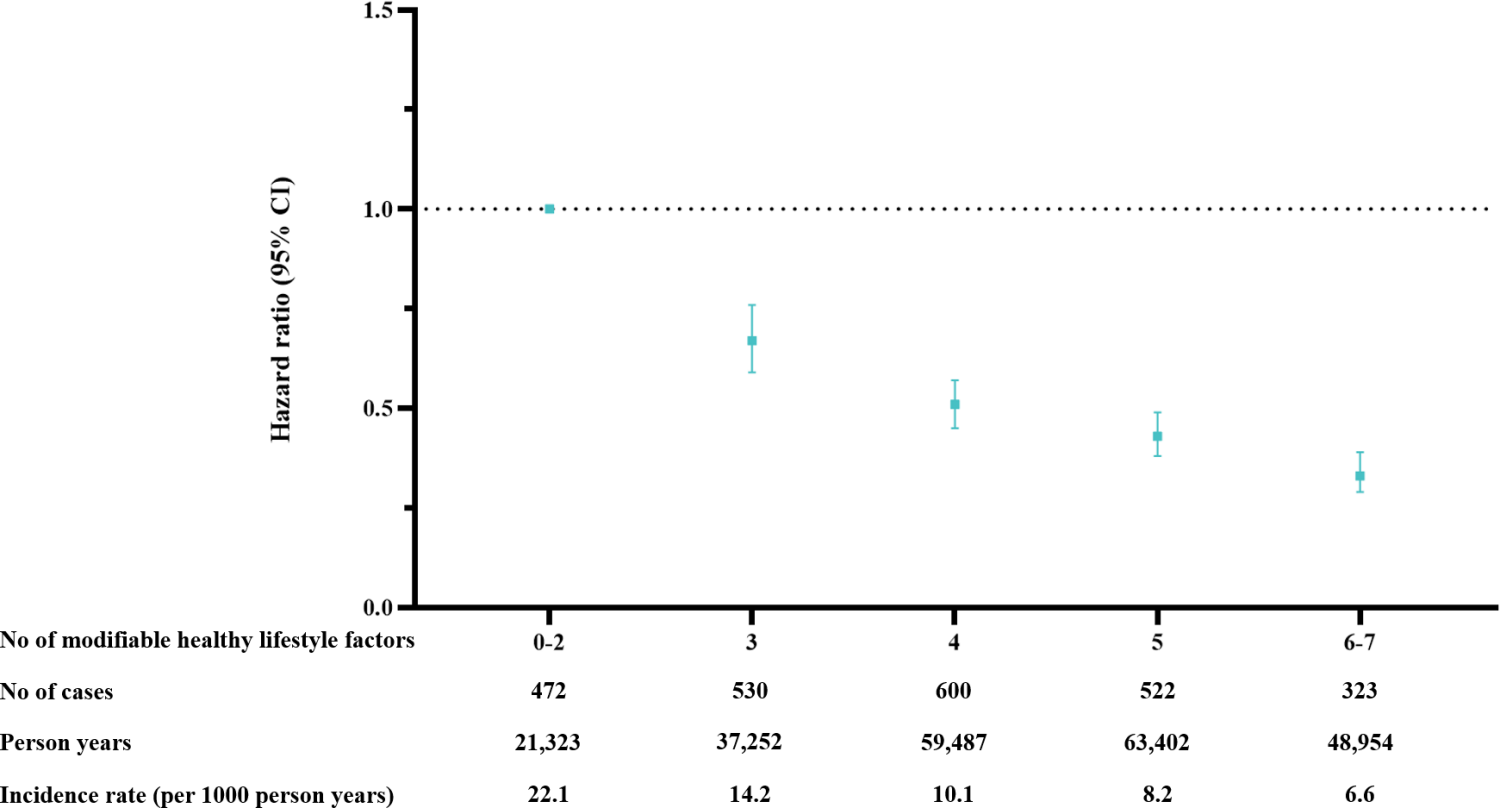
Hazard ratios of all-cause mortality according to combined modifiable healthy lifestyle factors.

For each participant, we calculated person-years of follow-up from the date of recruitment (between 2006 and 2010) to the date of death or the end of follow-up (December 2020 for England and Wales and November 2020 for Scotland and elsewhere), whichever occurred first. Cox proportional hazard models were applied to examine the associations of each lifestyle factor and the overall lifestyle score with all-cause and cause-specific mortality risk. The results were reported as hazard ratios (HRs) with 95% CIs. We assigned a median value to each lifestyle score category to test the linear trend. Kaplan-Meier survival analysis and curve were also applied to reveal the association between lifestyle score and the cumulative incidence of death (Figure 1). We adjusted for age, sex, education, Townsend deprivation index, and employment to control potential confounders. Besides, lifestyle factors were mutually adjusted for when the relation of each lifestyle factor to all-cause mortality was examined. In addition, we performed sex-stratified analyses to evaluate the relation of healthy lifestyle scores to all-cause mortality in men and women. We took the same approach to examine the relation of healthy lifestyle scores to all-cause mortality. The proportional hazards assumption was tested using a Schoenfeld residuals plot. The population-attributable risk (PAR), an estimate of the proportion of all-cause deaths that would theoretically have been prevented if all individuals had a lifestyle score of at least 3, 4, 5, or >6, was calculated, with the 0‐2 category as the reference group.

We conducted 2 sensitivity analyses to evaluate the robustness of our primary findings. First, we performed multiple imputations with chained equations with 5 datasets to deal with the missing values of exposure or covariates (Table S1 in Multimedia Appendix 1). Then, we excluded participants who died within 2 or 4 years of follow-up to reduce immortal bias (Table S2 in Multimedia Appendix 1).

*P* values were 2-sided with statistical significance set at less than .05. All analyses were performed using Stata SE (version 15; StataCorp).

## Results

### Population Characteristics

The baseline characteristics of 20,654 individuals with probable sarcopenia are shown in Table 1. The mean age of the population was 59.7 (SD 7.20) years, and 39.8% (n=8126) were men. The proportion of healthy lifestyle scores of 0‐2, 3, 4, 5, and 6‐7 was 9.7% (n=2015), 16.4% (n=3382), 25.7% (n=5316), 27.2% (n=5610), and 21% (n=4331), respectively. Participants with a lower lifestyle score (0‐2) were younger (mean 58.85, SD 7.15 vs 60.43, SD 7.11 y), less educated (20.8% (n=419) vs 51.5% (n=2232) with higher education level), less employed (19.1% (n=385) vs 42.3% (1833) employment rate), and more socioeconomically deprived (Townsend score: 1.27 vs −1.43), compared to high scorers (6-7). Conversely, higher lifestyle scorers (6-7) exhibited a lower prevalence of CVD [8.8% (n=383) vs 19.6% (n=394])], hypertension [33.7% (n=1460) vs 49.4% (n=996)], diabetes [7.5% (n=325) vs 15.4% (n=311)], hyperlipidemia [24.5% (n=1,061) vs 37.3% (n=752)], and depression [5.7% (n=245) vs 18.1% (n=364); Table 1].

**Table 1.** Baseline characteristics of participants with probable sarcopenia by lifestyle score category^a^.

Variables	Lifestyle score				
0‐2 (n=2015)	3 (n=3382)	4 (n=5316)	5 (n=5610)	6‐7 (n=4331)
Age, mean (SD)	58.85 (7.15)	59.70 (7.20)	59.84 (7.17)	60.12 (7.10)	60.43 (7.11)
Male, n (%)	874 (43.4)	1281 (37.9)	2046 (38.5)	2123 (37.8)	1802 (41.6)
Not current smoking, n (%)	1122 (55.7)	2816 (83.3)	4866 (91.5)	5393 (96.1)	4298 (99.2)
Moderate alcohol consumption, n (%)	216 (10.7)	570 (16.9)	1439 (27.1)	2003 (35.7)	2729 (63)
Diet >=5 recommended components, n (%)	246 (12.2)	1064 (31.5)	2587 (48.7)	3748 (66.8)	3884 (89.7)
Regular physical activity, n (%)	196 (9.7)	700 (20.7)	2008 (37.8)	3427 (61.1)	3772 (87.1)
Adequate sleep, n (%)	353 (17.5)	1211 (35.8)	2808 (52.8)	4039 (72)	3903 (90.1)
Never or short television watching time, n (%)	309 (15.3)	1129 (33.4)	2835 (53.3)	4099 (73.1)	3955 (91.3)
Not isolated social connection, n (%)	1034 (51.3)	2656 (78.5)	4721 (88.8)	5341 (95.2)	4264 (98.5)
Ethnicity, n (%)					
Asian	74 (3.7)	194 (5.7)	358 (6.7)	352 (6.3)	270 (6.2)
Black	42 (2.1)	54 (1.6)	76 (1.4)	75 (1.3)	42 (1)
Mixed	16 (0.8)	30 (0.9)	23 (0.4)	34 (0.6)	17 (0.4)
Other	39 (1.9)	36 (1.1)	77 (1.4)	80 (1.4)	47 (1.1)
White	1844 (91.5)	3068 (90.7)	4782 (90)	5069 (90.4)	3955 (91.3)
Education category, n (%)					
Higher education	419 (20.8)	930 (27.5)	1876 (35.3)	2367 (42.2)	2232 (51.5)
Upper secondary	110 (5.5)	188 (5.6)	353 (6.6)	405 (7.2)	276 (6.4)
Lower secondary	385 (19.1)	749 (22.1)	1222 (23)	1247 (22.2)	892 (20.6)
Vocational	131 (6.5)	231 (6.8)	316 (5.9)	307 (5.5)	214 (4.9)
Other or prefer not to answer	970 (48.1)	1284 (38)	1549 (29.1)	1284 (22.9)	717 (16.6)
Employment, n (%)	385 (19.1)	926 (27.4)	1918 (36.1)	2253 (40.2)	1833 (42.3)
Townsend, mean (SD)	1.27 (3.68)	0.01 (3.46)	−0.57 (3.30)	−1.17 (3.08)	−1.43 (2.90)
Conditions, n (%)					
Cancer	255 (12.7)	400 (11.8)	639 (12)	622 (11.1)	486 (11.2)
Cardiovascular disease	394 (19.6)	568 (16.8)	692 (13)	601 (10.7)	383 (8.8)
Hypertension	996 (49.4)	1520 (44.9)	2228 (41.9)	2100 (37.4)	1460 (33.7)
Diabetes mellitus	311 (15.4)	439 (13)	632 (11.9)	527 (9.4)	325 (7.5)
Hyperlipidemia	752 (37.3)	1112 (32.9)	1604 (30.2)	1529 (27.3)	1061 (24.5)
Neurodegenerative disease	24 (1.2)	32 (0.9)	45 (0.8)	25 (0.4)	18 (0.4)
Respiratory disease	251 (12.5)	226 (6.7)	226 (4.3)	200 (3.6)	104 (2.4)
Digestive disease	54 (2.7)	54 (1.6)	55 (1)	41 (0.7)	24 (0.6)
Depression	364 (18.1)	423 (12.5)	490 (9.2)	386 (6.9)	245 (5.7)

a Data are mean (SD) for continuous variables or n (%) for categorical variables.

### Association of Individual Lifestyle Factors With the Risk of All-Cause Mortality

During 230,418 person-years of follow-up, 2447 participants died. As shown in Table 2, each lifestyle factor was significantly associated with all-cause mortality. Compared with no current smoking participants, the adjusted HR of mortality was 1.98 (95% CI 1.78‐2.20) for those current smokers. Abstaining from smoking appears to be the most effective in reducing the risk of mortality. There was a U-shaped association between alcohol consumption and all-cause mortality, with moderate alcohol consumption (male ≤16 g per day; female ≤8 g per day regularly) being associated with lower mortality; other alcohol consumption levels (never or excessive) had a higher mortality risk (HR 1.28, 95% CI 1.17‐1.39). Engaging in irregular physical activity and following an unhealthy diet pattern were associated with a higher mortality rate; the corresponding HRs were 1.36 (95% CI 1.25‐1.47) and 1.11 (95% CI 1.02‐1.20). Adoption of 3 emerging healthy lifestyle factors was also associated with lower mortality risk. Short (≤6 h per day) or long duration (≥9 h per day) of sleep versus adequate sleeping duration (7‐8 h per day) was associated with a higher risk of mortality (HR 1.14, 95% CI 1.05‐1.24), especially the long sleep duration group had a significantly higher mortality rate (HR 1.39, 95% CI 1.25‐1.55; Table S3 in Multimedia Appendix 1). Participants with longer television-watching time (≥4 h per day) had higher mortality than those with short television-watching participants (HR 1.20, 95% CI 1.10‐1.31), and those who had an isolated social connection could also significantly increase the risk of all-cause mortality (HR 1.22, 95% CI 1.09‐1.35; Table 2). Besides, no apparent effect modification was observed by sex (Tables S4 and S5 in Multimedia Appendix 1).

**Table 2.** HR^a^ (95% CI) of all-cause mortality according to individual modifiable healthy lifestyle factors.

Modifiable healthy lifestyle factors^b^	Participants, n	Cases, n	Person-years	Follow-up time, mean (SD)	Incidence rate per 1000 person-year (95% CI)	HR (95% CI)^c^	*P* value
Smoking							<.001
No current	18,495	1966	207526.68	11.22 (1.91)	9.47 (9.06‐9.90)	1.00 (reference)	
Current	2159	481	22890.80	10.60 (2.75)	21.01 (19.18‐22.98)	1.98 (1.78‐2.20)	
Alcohol consumption							<.001
Moderate	6957	785	77110.68	11.08 (1.92)	10.18 (9.48‐10.92)	1.00 (reference)	
Never or excessive	13,697	1662	153306.80	11.19 (2.07)	10.84 (10.33‐11.38)	1.28 (1.17‐1.39)	
Diet							.01
>=5 recommended components	11,529	1205	129681.51	11.25 (1.83)	9.29 (8.77‐9.83)	1.00 (reference)	
<5 recommended components	9125	1242	100735.97	11.04 (2.24)	12.33 (11.65‐13.03)	1.11 (1.02‐1.20)	
Physical activity							<.001
Regular	10,103	978	113630.74	11.25 (1.80)	8.61 (8.08‐9.16)	1.00 (reference)	
Irregular	10,551	1469	116786.74	11.07 (2.21)	12.58 (11.94‐13.24)	1.36 (1.25‐1.47)	
Sleep							.001
Adequate	12,314	1307	138003.41	11.21 (1.92)	9.47 (8.96‐10.00)	1.00 (reference)	
Short or long	8340	1140	92414.07	11.08 (2.17)	12.34 (11.63‐13.07)	1.14 (1.05‐1.24)	
Television watching time							<.001
Short	12,327	1161	138817.19	11.26 (1.81)	8.36 (7.89‐8.86)	1.00 (reference)	
Long	8327	1286	91600.29	11.00 (2.29)	14.04 (13.28‐14.83)	1.20 (1.10‐1.31)	
Social connection							<.001
Appropriate	18,016	1994	201699.16	11.20 (1.96)	9.89 (9.46‐10.33)	1.00 (reference)	
Isolated	2638	453	28718.32	10.89 (2.41)	15.77 (14.35‐17.30)	1.22 (1.09‐1.35)	

a HR: hazard ratio.

b Low-risk lifestyle factors: no current smoking, moderate alcohol consumption (must be drinking but no more than 1 drink per day for women and 2 drinks per day for men on a relatively regular frequency, no drinking is risk factor), healthy diet (adequate intake of at least one-half of 10 recommended food groups), regular physical activity (150 min per week of moderate activity or 75 min per week of vigorous activity, or an equivalent combination), adequate sleep duration (7-8 h per day), short television watching time (<4 h per day), and appropriate social connection (not isolated).

c Adjustment for age (years), sex (women or men), ethnicity (White, Black, Asian, Mixed, or other), education (higher, ie, college or university degree or other professional qualification; upper secondary, ie, second or final stage of secondary education; lower secondary, ie, first stage of secondary education; vocational, ie, work-related practical qualifications; or other), Townsend deprivation index, and employment (currently employed or not). Lifestyle factors were mutually adjusted for analyses on the association of each individual lifestyle factor with all-cause mortality risk.

### Association of Lifestyle Score With Risk of All-Cause Mortality

**Figure 2.**
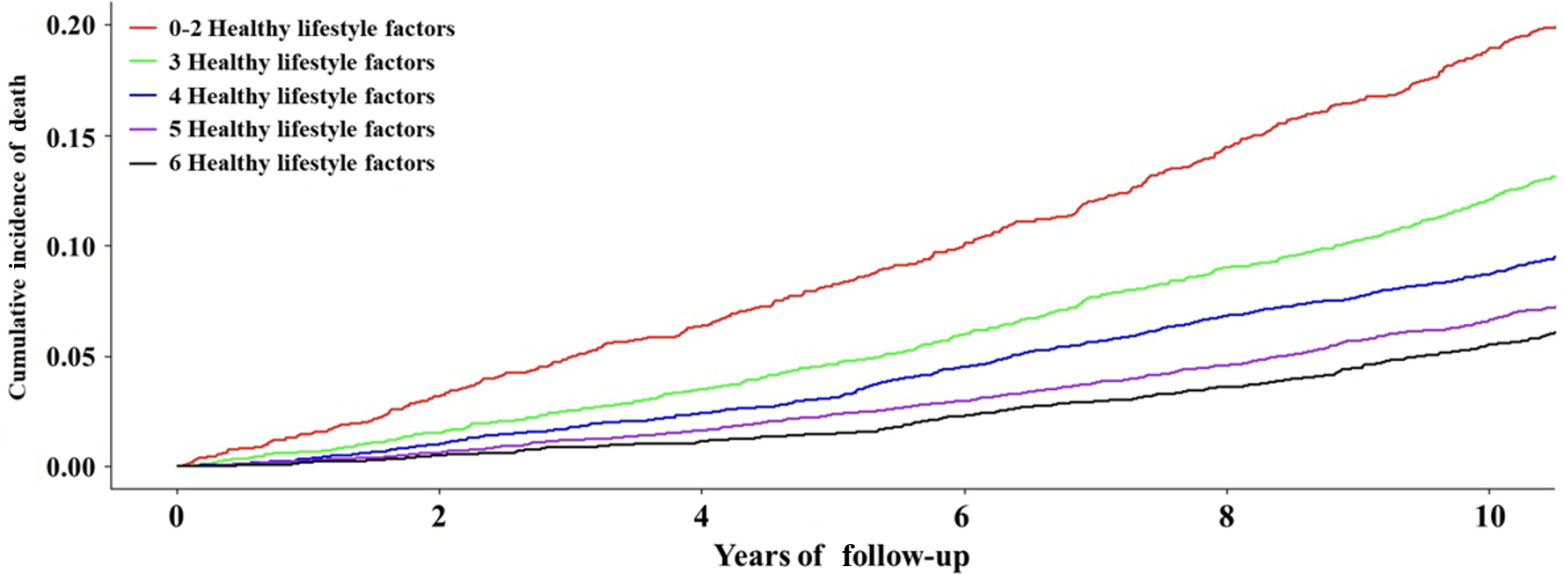
Kaplan-Meier survival curve of all-cause mortality according to combined modifiable healthy lifestyle factors.

The healthy lifestyle score was inversely associated with all-cause mortality (Table 3). For each one-point increase, the HR of all-cause mortality risk was 0.78 (95% CI 0.76‐0.81; *P* for trend <.001). Compared with those scoring 0‐2, the multivariable-adjusted HRs of mortality for participants with healthy lifestyle scores of 3, 4, 5, and 6‐7 were 0.67 (95% CI 0.59‐0.76), 0.51 (95% CI 0.45‐0.57), 0.43 (95% CI 0.38‐0.49), and 0.33 (95% CI 0.29‐0.39), respectively (*P* for trend <.001; Figure 2). The PAR for all-cause mortality increased as the healthy lifestyle score increased, from 3.1% for a score of ≥2% to 9.7%, 18.3%, 25.7%, and 34.6% for scores of ≥3, ≥4, ≥5, and ≥6, respectively (Table 4).

**Table 3.** HR^a^ (95% CI) of all-cause or cause-specific mortality according to combined modifiable healthy lifestyle factors.

Variables	Number of modifiable healthy lifestyle factors^b^					*P* value	HR of each point increase (95% CI)
	0‐2 (n=2015)	3 (n=3382)	4 (n=5316)	5 (n=5610)	6‐7 (n=4331)		
All-cause mortality						<.001	0.78 (0.76‐0.81)
Number of cases	472	530	600	522	323		
Person-years	21,323	37,252	59,487	63,402	48,954		
HR (95% CI)	1.00 (reference)	0.68 (0.60‐0.77)	0.51 (0.45‐0.57)	0.43 (0.37‐0.49)	0.33 (0.28‐0.38)		
Cancer						<.001	0.82 (0.78‐0.86)
Number of cases	172	218	245	207	151		
Person-years	21,323	37,252	59,487	63,402	48,954		
HR (95% CI)	1.00 (reference)	0.76 (0.62‐0.94)	0.57 (0.47‐0.70)	0.46 (0.37‐0.57)	0.43 (0.34‐0.55)		
Cardiovascular disease						<.001	0.83 (0.77‐0.88)
Number of cases	100	111	142	114	66		
Person-years	21,323	37,252	59,487	63,402	48,954		
HR (95% CI)	1.00 (reference)	0.71 (0.54‐0.94)	0.65 (0.50‐0.85)	0.53 (0.40‐0.71)	0.39 (0.28‐0.55)		
Respiratory disease						<.001	0.73 (0.66‐0.80)
Number of cases	74	62	48	47	31		
Person-years	21,323	37,252	59,487	63,402	48,954		
HR (95% CI)	1.00 (reference)	0.57 (0.41‐0.81)	0.31 (0.21‐0.45)	0.31 (0.21‐0.45)	0.26 (0.17‐0.41)		
Neurodegenerative disease						.08	0.89 (0.95‐1.02)
Number of cases	18	25	37	44	32		
Person-years	21,323	37,252	59,487	63,402	48,954		
HR (95% CI)	1.00 (reference)	0.70 (0.38‐1.29)	0.61 (0.35‐1.08)	0.63 (0.36‐1.10)	0.55 (0.30‐0.98)		
Digestive disease						<.001	0.66 (0.58‐0.75)
Number of cases	43	37	28	27	8		
Person-years	21,323	37,252	59,487	63,402	48,954		
HR (95% CI)	1.00 (reference)	0.60 (0.39‐0.94)	0.33 (0.20‐0.53)	0.32 (0.19‐0.52)	0.12 (0.06‐0.27)		
Other^c^						<.001	0.81 (0.75‐0.88)
Number of cases	65	77	100	83	35		
Person-years	21,323	37,252	59,487	63,402	48,954		
HR (95% CI)	1.00 (reference)	0.79 (0.56‐1.10)	0.71 (0.51‐0.98)	0.59 (0.41‐0.84)	0.33 (0.21‐0.51)		

a HR: hazard ratio.

b Adjustment for age (years), sex (women or men), ethnicity (White, Black, Asian, Mixed or other), education (higher, ie, college or university degree or other professional qualification; upper secondary, ie, second or final stage of secondary education; lower secondary, ie, first stage of secondary education; vocational, ie, work-related practical qualifications; or other), Townsend deprivation index, and employment (currently employed or not); adjusting for competing risk of death of other causes.

c Mortality from causes other than cancer, CVD, respiratory disease, neurodegenerativee disease, or digestive disease.

**Table 4.** Relative and population-attributable risks of all-cause mortality for groups defined by combinations of modifiable healthy lifestyle factors.

Modifiable healthy lifestyle factors^a^	Number of cases	% of total population	Hazard ratio (95% CI)^b^	PAR%^c^ (95% CI)	
At least 2 vs <2	2309	94.4	0.46 (0.38‐0.55)	3.1 (2.6‐3.5)	
At least 3 vs <3	1975	80.7	0.50 (0.45‐0.55)	9.7 (8.6‐10.7)	
At least 4 vs <4	1445	59.1	0.55 (0.51‐0.60)	18.3 (16.3‐20.2)	
At least 5 vs <5	845	34.5	0.61 (0.56‐0.66)	25.7 (22.0‐29.0)	
At least 6 vs <6	323	13.2	0.60 (0.53‐0.68)	34.6 (27.9‐40.5)	

a Low-risk lifestyle factors: no current smoking, moderate alcohol consumption (no more than 1 drink per day for women and 2 drinks per day for men on a relatively regular frequency), healthy diet (adequate intake of at least one-half of 10 recommended food groups), regular physical activity (150 min per week of moderate activity or 75 min per week of vigorous activity, or an equivalent combination), adequate sleep duration (7-8 h per day), short television watching time (<4 h per day), and appropriate social connection (not isolated).

b Adjustment for age (years), sex (women or men), ethnicity (White, Black, Asian, Mixed, or other), education (higher, ie, college or university degree or other professional qualification; upper secondary, ie, second or final stage of secondary education; lower secondary, ie, first stage of secondary education; vocational, ie, work-related practical qualifications; or other), Townsend deprivation index, and employment (currently employed or not).

c PAR%: population attributable risk percent.

HRs and their 95% CIs were calculated in Cox proportional hazard models after adjusting for age (years), sex (women or men), ethnicity (White, Black, Asian, Mixed or other), education (higher, ie, college or university degree or other professional qualification; upper secondary, ie, second or final stage of secondary education; lower secondary, ie, first stage of secondary education; vocational, ie, work-related practical qualifications; or other), Townsend deprivation index, and employment (currently employed or not).

### Association of Lifestyle Score With Risk of Cause-Specific Mortality

During the follow-up, 993 participants died from cancer, 533 participants died from CVD, 262 participants died from respiratory disease, 156 participants died from neurodegenerative disease, 143 participants died from digestive disease, and 360 participants died from other causes. There was a significantly inverse dose-response association between the health lifestyle score and the mortality from cancer, CVD, respiratory disease, digestive disease, and other causes (*P* for trend <.001); however, no such pattern was observed for neurodegenerative disease (Table 3). Participants with higher overall lifestyle scores (6-7) had significantly lower mortality rates compared to those with scores of 0‐2. The multivariate-adjusted HRs of cause-specific mortality were 0.43 (95% CI 0.34‐0.55) for cancer, 0.39 (95% CI 0.28‐0.55) for CVD, 0.26 (95% CI 0.17‐0.41) for respiratory disease, 0.12 (95% CI 0.06‐0.27) for digestive disease, and 0.33 (95% CI 0.21‐0.51) for other causes.

## Discussion

### Statement of Principal Findings

In this cohort study conducted among individuals with probable sarcopenia, we found that adopting a healthy lifestyle was associated with significantly reduced all-cause mortality, notably deaths from cancer, CVD, respiratory disease, and digestive disease. We estimated that approximately 25.7% of deaths could be prevented if they could adopt six or more healthy lifestyle factors. These findings underscore the pivotal role of embracing a healthy lifestyle in alleviating mortality among individuals with probable sarcopenia.

### Comparison With Previous Studies

Physical activity and nutritional interventions have already been recommended as primary strategies for managing sarcopenia, but previous evidence was inconclusive, with limitations such as small sample sizes and inconsistent adherence to interventions [28,29]. This study provides further evidence for the effectiveness of these two interventions among individuals with probable sarcopenia. Meanwhile, this study highlights divergent associations between lifestyle factors and mortality in probable sarcopenia populations versus the general population. Among individuals with probable sarcopenia, current smoking and excessive alcohol consumption exert the strongest mortality impacts (Table S3 in Multimedia Appendix 1). By contrast, in the general population, smoking cessation remains the most impactful lifestyle determinant on mortality [30], and Locquet et al [31] also discovered that smokers have a 2.36-fold higher likelihood of developing sarcopenia. Regular physical activity conferred the second-greatest survival benefit in the general population [30], which was consistent with prior reports by Li et al [32]; while dietary quality (HR 0.88, 95% CI 0.82‐0.94 in men; HR 0.97, 95% CI 0.90‐1.05 in women) and excessive alcohol intake (HR 0.95, 95% CI 0.89‐1.01 in men; HR 1.03, 95% CI 0.94‐1.12 in women) had weaker associations with mortality [30]. Notably, a meta-analysis also indicated that alcohol consumption is not associated with the presence of sarcopenia in the general population, with a pooled odds ratio of 0.964 [33]. Overall, these results indicate that it is meaningful to explore the impact of these healthy lifestyle factors on the mortality risk within the specific population of probable sarcopenia.

Three emerging healthy lifestyle factors also show a significant association with reduced risk of mortality. Social connection has garnered significant attention since the onset of the COVID-19 pandemic [34]. Previous studies have reported that individuals with sarcopenia are more prone to experiencing social isolation [17]. This study, for the first time, provides evidence for the beneficial effects of social connection on the probable sarcopenia population. Additionally, a recent meta-analysis showed that both short and long sleep durations are associated with an increased risk of all-cause mortality and cardiovascular events in the general population [35]. In this study, however, only long sleep duration was significantly associated with an increased mortality risk. There are two possible reasons: (1) individuals with probable sarcopenia who spend less time on sleep may allocate more time for physical activities, the interconnectedness among different lifestyle factors thereby potentially influencing the underlying association between lifestyle factors and mortality risk; and (2) sleep quality, such as chronotype, insomnia, snoring, and daytime sleepiness, can also affect the health outcomes [36].

To our knowledge, no study has examined the association between the combined healthy lifestyle score and mortality risk among individuals with sarcopenia. We have used Cox proportional hazard models and PAR to uncover whether and to what extent individuals with probable sarcopenia can benefit from a healthy lifestyle. These findings indicate that, beyond physical activity and diet, other healthy lifestyle factors also confer benefits to the probable sarcopenia population. This knowledge can contribute to the development of “core set outcomes” for lifestyle-based intervention trials in the field of sarcopenia. Additionally, a higher healthy lifestyle score is also associated with a reduced risk of mortality due to cancer, CVD, respiratory disease, and digestive disease, although not for neurodegenerative disease. Previous studies have demonstrated that adopting a healthy lifestyle can lower the risk of developing neurodegenerative diseases [37]. Nevertheless, since neurodegenerative diseases are typically not immediately life-threatening, their specific mortality risk might be attenuated.

### Potential Mechanisms

Biological mechanisms linking various lifestyle factors to health outcomes have been postulated. Physical activity can impact systemic immune and metabolic response, stimulating adenosine monophosphate-activated protein kinase phosphorylation or other critical signaling pathways to promote multiple organ health and increase survival probability [38-40]. A balanced diet can provide sufficient protein, amino acids, and other essential nutrients [29]. Additionally, it can also help maintain intestinal microflora homeostasis and influence the aging gut, extending life span through metabolites produced by gut microbiota, such as short-chain fatty acids [41].

Cigarettes contain many toxic and carcinogenic components, which can impair mitochondrial function, increase oxidative stress [42], and cause epigenetic changes [43]. Therefore, avoiding tobacco exposure is an effective measure to lower disease and mortality risks. Individuals who frequently consume moderate amounts of alcohol show a lower mortality risk than those who drink alcohol rarely or excessively. A possible explanation for this phenomenon may lie in the fact that individuals who engage in regular moderate drinking tend to have better financial situations and social connections. Mechanistically, moderate alcohol consumption can lower the activity of a mental stress–related brain network [44] and reduce the risk of major cardiovascular events [45]. Besides, excessive drinking leads to inflammation and immune-metabolic dysregulation, gut leak and dysbiosis, mitochondrial dysfunction, and epigenomic modifications [46-48]. These mechanisms synergistically interact to cause alcohol-mediated multiorgan injury and even death.

Sedentary behavior is directly associated with elevated levels of circulating inflammatory markers, such as interleukin-6 and C-reactive protein [49,50]. These markers have been proposed as senescent biomarkers due to their positive correlation with age and potential promotion of adverse health outcomes in older adults [51]. Growing evidence indicates that social connection influences various biological pathways, including blood pressure [52], oxidative stress [53], neuroendocrine dysregulation [54], chronic inflammation [55], and gut-microbiome interactions [56]. Similarly, social connections may indirectly influence health outcomes via stress perception and other behavioral factors, such as sleep quality and quantity, smoking, or even drug abuse [57,58]. These social stress-related behaviors are strongly associated with biological health.

Sarcopenia is associated with the aforementioned pathologic mechanisms that drive increased mortality risk and are influenced by lifestyle [59,60]. Individuals with sarcopenia often have multiple comorbidities (eg, diabetes, kidney disease, and chronic obstructive pulmonary disease) [61,62], and evidence indicates that healthy lifestyles significantly aid in managing these conditions [63,64], thereby reducing mortality risk.

### Clinical and Research Implications

First, our findings provide incentive and support for the notion that lifestyle intervention strategies can have an impressive impact on mortality risk among individuals with probable sarcopenia, and this could help health professionals and policy makers to make preventive advice and policy recommendations for this population. Second, this study presented precise numerical values to which individuals with probable sarcopenia can benefit from a healthy lifestyle. Such knowledge might help motivate individuals with probable sarcopenia to change habits and adhere to a healthy lifestyle. Third, only 21% of the participants adhered to a healthy lifestyle (6‐7 healthy factors) in this study. In comparison, 51.9% adopted less than or equal to four healthy lifestyle factors, suggesting a large gap between current and ideal population lifestyles. Last but not least, this study found that certain lifestyle factors are more relevant than others; therefore, public health policies could focus on a few more potent risk factors (ie, smoking) rather than on costly strategies addressing multiple risk factors.

### Strengths and Limitations

This study has several strengths. Foremost among these is the large sample size and comprehensive data resources, which encompassed detailed information on potential confounding variables. This robust dataset facilitated thorough analyses of mortality across various causes and allowed for stratification based on potential risk factors [22]. Additionally, this study pioneered the assessment of the combined impact of an overall healthy lifestyle on the probable sarcopenia population. This approach was particularly important due to the strong intercorrelations among various lifestyle factors [65]. Using an overall healthy lifestyle score enabled a comprehensive evaluation of the intricate relationships between lifestyle factors and mortality among individuals with probable sarcopenia.

However, several limitations should be acknowledged. First, the study’s participant pool primarily consisted of Caucasians in the United Kingdom. Consequently, the generalizability of our findings to other ethnic groups may be limited. Second, due to the nature of the observational study, we cannot derive causality between lifestyle modification and mortality in this population, which warrants more well-conducted interventional studies to verify. Third, the temporal relationship between lifestyle factors and probable sarcopenia could not be clearly demarcated in this study. Thus, potential collider bias may arise when conditioning on probable sarcopenia status in analyses of lifestyle factors and mortality. This residual collider bias could attenuate associations toward the null [66], yet our identification of strong relationships strengthens confidence in these conclusions. Given this bias, estimating potentially preventable deaths via postprobable sarcopenia lifestyle changes would better clarify the importance of lifestyle management. However, UK Biobank’s limited longitudinal data on postdiagnosis lifestyle modifications precluded formal analysis of how such changes affect mortality [19]. Fourth, further research can be conducted on confirmed patients with sarcopenia, although investigating lifestyles’ impact on the risk of premature mortality among the probable sarcopenia population holds greater preventive implications. Fourth, measurement errors could occur in the self-reported lifestyle data. Fifth, those who died during the study period might have had serious diseases at baseline. Although the study excluded deaths within the first 2‐4 years of follow-up, the possibility of reverse causation and residual confounding remains. Finally, even though various covariates were adjusted for in our analyses, other confounders, such as BMI and comorbidities, may not have been included, which could result in residual confounding.

### Conclusions

A healthy lifestyle was associated with a lower risk of all-cause mortality and mortality due to cancer, CVD, respiratory disease, and digestive disease among individuals with probable sarcopenia. A healthy lifestyle (scoring 6‐7) could prevent 25.7% of deaths in this population.

## Supplementary material

10.2196/65374 Multimedia Appendix 1 Supplemental methods and results.

## Abbreviations

CVD: cardiovascular disease
EWGSOP2: European Working Group on Sarcopenia in Older People 2
HR: hazard ratio
PAR: population-attributable risk
