# Supplementary material for: Association of a Healthy Lifestyle With All-Cause and Cause-Specific Mortality Among Individuals With Probable Sarcopenia: Population-Based Cohort Study
Source: JMIR Aging. 2025 Jul 28;8:e65374. doi: 10.2196/65374 (PMC12303552; doi:10.2196/65374)
Supplement: Multimedia Appendix 1 [file aging-v8-e65374-s001.docx]

**Web Appendix**

**Association of a healthy lifestyle with all-cause and cause-specific mortality among individuals with sarcopenia: a population-based cohort study**

**Table of Contents**

[*Supplemental methods* 2](#_Toc203751777)

[**Table S1.** Hazard ratio (95% CI) of cause specific mortality according to combined modifiable healthy lifestyle factors using multiple imputations with chained equations to assign missing values of exposure and covariates 4](#_Toc203751778)

[**Table S2.** Hazard ratio (95% CI) of cause specific mortality according to combined modifiable healthy lifestyle fact 、ors, excluding deaths that occurred within the first 2 or 4 years of follow-up 6](#_Toc203751779)

[**Table S3.** Hazard ratio (95% CI) of all-cause mortality according to individual modifiable healthy lifestyle factors 9](#_Toc203751780)

[**Table S4.** Hazard ratio (95% CI) of all-cause mortality according to individual modifiable healthy lifestyle factors in male (binary variable) 10](#_Toc203751781)

[**Table S5.** Hazard ratio (95% CI) of all-cause mortality according to individual modifiable healthy lifestyle factors in female (binary variable) 12](#_Toc203751782)

*Supplemental methods*

Assessment of lifestyle factors

All information on lifestyle factors was self-reported using a touchscreen questionnaire at baseline (2006-2010). Detailed questionnaires could be obtained through the UK Biobank website (https://biobank.ndph.ox.ac.uk/showcase/).

- Smoking: Participants were asked about their current smoking status through the following question: “Do you smoke tobacco now? i. yes, on most or all days, ii. only occasionally, iii. no, or iv. prefer not to answer”. Except for those who indicated they were currently smoking on most or all days, participants were further asked about their past smoking frequency through “In the past, how often have you smoked tobacco? i. smoked on most or all days, ii. smoked occasionally, iii. just tried once or twice, iv. I have never smoked, or v. prefer not to answer”. The smoking status was then categorized into three groups (current, previous, and never).
- Alcohol consumption: Participants were asked about their drinking frequency through the following question: “About how often do you drink alcohol? i. daily or almost daily, ii. three or four times a week, iii. once or twice a week, iv. one to three times a month, v. special occasions only, vi. never, or vii. prefer not to answer”. Depending on their reported frequency of alcohol drinking, participants were further asked how much red wine (glasses), champagne plus white wine (glasses), beer plus cider (pints), spirits (measures), fortified wine (glasses), and other alcoholic drinks (glasses) they drank on average in a week or month. We calculated the average amount of daily alcohol intake of each participant based on a previous UK Biobank study. (1) Alcohol consumption status was defined by integrated information on drinking frequency and estimated intake total.
- Physical activity: The number of days of moderate/vigorous physical activity was recorded by asking “In a typical week, on how many days did you do 10 minutes or more of moderate physical activities like carrying light loads, cycling at a normal pace? (not include walking)” and “In a typical week, how many days did you do 10 minutes or more of vigorous physical activity? (These are activities that make you sweat or breathe hard such as fast cycling, aerobics, heavy lifting)”. According to their reported number of days, participants were further asked about the duration of moderate/vigorous physical activity through the following questions: “How many minutes did you usually spend doing moderate activities on a typical day?” and “How many minutes did you usually spend doing vigorous activities on a typical day?”. We multiplied the number of days and the duration per day to calculate the weekly total amount of moderate or vigorous physical activity, respectively.
- Diet: According to a previous UK Biobank study, (2) we selected 10 food groups that have been recommended as dietary priorities for cardiometabolic health (3) to evaluate diet qualities. A healthy diet was defined as an adequate intake of at least half of the 10 recommended food groups. Details were shown as follows:

| **Diet Component** | **Intake Goal** | **Field IDs** |
| --- | --- | --- |
| Fruits | ≥3 servings/day | 1309-"About how many pieces of FRESH fruit would you eat per DAY?" |
|  |  | 1319-"About how many pieces of DRIED fruit would you eat per DAY?" |
| Vegetables | ≥3 servings/day | 1289-"On average how many heaped tablespoons of COOKED vegetables would you eat per DAY?" |
|  |  | 1299-"On average how many heaped tablespoons of SALAD or RAW vegetables would you eat per DAY?" |
| Whole grains | ≥3 servings/day | 1438-"How many slices of bread do you eat each WEEK?" |
|  |  | 1448-"What type of bread do you mainly eat?" |
|  |  | 1458-"How many bowls of cereal do you eat a WEEK?" |
|  |  | 1468-"What type of cereal do you mainly eat?" |
| (Shell)fish | ≥2 servings/week | 1329-"How often do you eat oily fish? (e.g. sardines, salmon, mackerel, herring)" |
|  |  | 1339-"How often do you eat other types of fish? (e.g. cod, tinned tuna, haddock)" |
| Dairy | ≥2 servings/day | 1408-"How often do you eat cheese? (Include cheese in pizzas, quiches, cheese sauce etc.)" |
|  |  | 1418-"What type of milk do you mainly use?" |
| Vegetable oils | ≥2 servings/day | 1428-"What type of spread do you mainly use?" (Spread type) |
|  |  | 2654-"What type of spread do you mainly use?" (Non-butter spread type details) |
|  |  | 1438-"How many slices of bread do you eat each WEEK?" |
| Refined grains | ≤2 servings/day | 1438-"How many slices of bread do you eat each WEEK?" |
|  |  | 1448-"What type of bread do you mainly eat?" |
|  |  | 1458-"How many bowls of cereal do you eat a WEEK?" |
|  |  | 1468-"What type of cereal do you mainly eat?" |
| Processed meats | ≤1 serving/week | 1349-"How often do you eat processed meats (such as bacon, ham, sausages, meat pies, kebabs, burgers, chicken nuggets)" |
| Unprocessed meats | ≤2 servings/week | 1359-"How often do you eat chicken, turkey or other poultry? (Do not count processed meats)" |
|  |  | 1369-"How often do you eat beef? (Do not count processed meats)" |
|  |  | 1379-"How often do you eat lamb/mutton? (Do not count processed meats)" |
|  |  | 1389-"How often do you eat pork? (Do not count processed meats such as bacon or ham)" |
| Sugar-sweetened beverages | Don’t drink | 6144-"Which of the following do you NEVER eat?" |

- Sleep duration: Total sleep duration was recorded as the number of reported hours of sleep by asking “About how many hours sleep do you get in every 24 hours? (Please include naps)”.
- Television watching time: Time spent watching television was recorded as the number of hours watching television by asking “In a typical DAY, how many hours do you spend watching TV? (Put 0 if you do not spend any time doing it)”.
- Social connection: Participants were asked about their social connection status through the following three questions: [1] “Including yourself, how many people are living together in your household? Include those who usually live in the house such as students living away from home during term time, partners in the armed forces or professions such as pilots”; [2] “How often do you visit friends or family or have them visit you? i. almost daily, ii. 2-4 times a week, iii. about once a week, iv. about once a month, v. once every few months, vi. never or almost never, vii. no friends/family outside household, viii. do not know, or ix. prefer not to answer”; [3] “Which of the following [leisure/social activities] do you attend once a week or more often? You can select more than one: i. sports club or gym, ii. pub or social club, iii. religious group, iv. adult education class, v. other group activity, vi. none of the above, vii. prefer not to answer”. According to a previous UK Biobank study, (4) we assigned 1 point to participants reporting “living alone”, 1 point to those reporting “friends and family visit less than once a month”, and 1 point to those reporting “no participation in social activities at least weekly”. Individual scores were summed up to calculate an overall social score ranging from 0-3. We then defined the social connection status as “active” (score=0), “moderately active” (score=1), and “isolated” (score≥2) based on the social score.

**Table S1.** Hazard ratio (95% CI) of cause specific mortality according to combined modifiable healthy lifestyle factors using multiple imputations with chained equations to assign missing values of exposure and covariates

|  | **Number of Modifiable Healthy Lifestyle Factors^a^** | | | | | **p for trend** | **HR of each point increase** |
| --- | --- | --- | --- | --- | --- | --- | --- |
|  | **0-2 (n=3,193)** | **3 (n=4,660)** | **4 (n=6,941)** | **5 (n=6,846)** | **6-7 (n=5,133)** |  |  |
| **All cause Mortality** |  |  |  |  |  |  |  |
| No. of cases/person-years | 720/33,951 | 765/51,298 | 844/77,580 | 650/77,401 | 406/57,930 |  |  |
| Hazard ratio (95% CI) | 1.00 | 0.74 (0.67-0.82) | 0.57 (0.51-0.63) | 0.46 (0.41-0.51) | 0.38 (0.33-0.43) | <0.001 | 0.80 (0.78-0.82) |
| **Cancer** |  |  |  |  |  |  |  |
| No. of cases/person-years | 255/33,951 | 294/51,298 | 330/77,580 | 249/77,401 | 188/57,930 |  |  |
| Hazard ratio (95% CI) | 1.00 | 0.79 (0.67-0.94) | 0.62 (0.52-0.73) | 0.48 (0.40-0.58) | 0.49 (0.40-0.59) | <0.001 | 0.83 (0.80-0.87) |
| **CVD** |  |  |  |  |  |  |  |
| No. of cases/person-years | 160/33,951 | 176/51,298 | 205/77,580 | 149/77,401 | 83/57,930 |  |  |
| Hazard ratio (95% CI) | 1.00 | 0.83 (0.66-1.03) | 0.70 (0.57-0.88) | 0.56 (0.44-0.71) | 0.42 (0.31-0.56) | <0.001 | 0.83 (0.79-0.88) |
| **Respiratory disease** |  |  |  |  |  |  |  |
| No. of cases/person-years | 110/33,951 | 87/51,298 | 74/77,580 | 63/77,401 | 40/57,930 |  |  |
| Hazard ratio (95% CI) | 1.00 | 0.60 (0.45-0.79) | 0.38 (0.28-0.51) | 0.35 (0.26-0.49) | 0.31 (0.21-0.45) | <0.001 | 0.76 (0.70-0.82) |
| **Neurodegenerative disease** |  |  |  |  |  |  |  |
| No. of cases/person-years | 29/33,951 | 46/51,298 | 51/77,580 | 57/77,401 | 38/57,930 |  |  |
| Hazard ratio (95% CI) | 1.00 | 0.93 (0.58-1.49) | 0.64 (0.40-1.01) | 0.67 (0.42-1.07) | 0.56 (0.34-0.93) | 0.011 | 0.88 (0.79-0.97) |
| **Digestive disease** |  |  |  |  |  |  |  |
| No. of cases/person-years | 60/33,951 | 46/51,298 | 39/77,580 | 30/77,401 | 8/57,930 |  |  |
| Hazard ratio (95% CI) | 1.00 | 0.60 (0.41-0.89) | 0.38 (0.25-0.58) | 0.32 (0.20-0.50) | 0.12 (0.05-0.24) | <0.001 | 0.66 (0.60-0.74) |
| **Other^b^** |  |  |  |  |  |  |  |
| No. of cases/person-years | 106/33,951 | 116/51,298 | 145/77,580 | 102/77,401 | 49/57,930 |  |  |
| Hazard ratio (95% CI) | 1.00 | 0.84 (0.64-1.10) | 0.77 (0.59-1.00) | 0.60 (0.45-0.81) | 0.41 (0.28-0.59) | <0.001 | 0.84 (0.78-0.89) |

Abbreviations: CI, confidence interval.

^a^Adjustment for age (years), sex (women or men), ethnicity (White, Black, Asian, Mixed or other), education {higher (college/university degree or other professional qualification), upper secondary (second/final stage of secondary education), lower secondary (first stage of secondary education), vocational (work-related practical qualifications), or other}, Townsend deprivation index, and employment (currently employed or not); adjusting for competing risk of death of other causes.

^b^Mortality from causes other than cancer, CVD, respiratory disease, neurogenerative disease, or digestive disease.

**Table S2.** Hazard ratio (95% CI) of cause specific mortality according to combined modifiable healthy lifestyle factors, excluding deaths that occurred within the first 2 or 4 years of follow-up

|  | **Number of Modifiable Healthy Lifestyle Factors^a^** | | | | | **p for trend** | **HR of each point increase** |
| --- | --- | --- | --- | --- | --- | --- | --- |
|  | **0-2** | **3** | **4** | **5** | **6-7** |  |  |
| **Excluding deaths that occurred within the first 2 years of follow-up** | | | | | | | |
|  | **N=1,951** | **N=3,330** | **N=5,262** | **N=5,576** | **N=4,309** |  |  |
| **All cause Mortality** | | | | | | |  |
| No. of cases/person-years | 408/21,256 | 478/37,196 | 546/59,422 | 488/63,364 | 301/48,925 |  |  |
| Hazard ratio (95% CI) | 1 | 0.69 (0.61-0.79) | 0.52 (0.46-0.60) | 0.45 (0.39-0.52) | 0.35 (0.30-0.41) | <0.001 | 0.80 (0.77-0.82) |
| **Cancer** |  |  |  |  |  |  |  |
| No. of cases/person-years | 143/21,256 | 193/37,196 | 219/59,422 | 192/63,364 | 141/48,925 |  |  |
| Hazard ratio (95% CI) | 1 | 0.80 (0.64-1.00) | 0.60 (0.48-0.74) | 0.50 (0.40-0.63) | 0.48 (0.37-0.61) | <0.001 | 0.84 (0.80-0.88) |
| **CVD** |  |  |  |  |  |  |  |
| No. of cases/person-years | 85/21,256 | 100/37,196 | 124/59,422 | 109/63,364 | 58/48,925 |  |  |
| Hazard ratio (95% CI) | 1 | 0.73 (0.54-0.98) | 0.63 (0.47-0.84) | 0.56 (0.41-0.76) | 0.38 (0.26-0.54) | <0.001 | 0.82 (0.77-0.88) |
| **Respiratory disease** |  |  |  |  |  |  |  |
| No. of cases/person-years | 70/21,256 | 57/37,196 | 45/59,422 | 43/63,364 | 31/48,925 |  |  |
| Hazard ratio (95% CI) | 1 | 0.54 (0.38-0.77) | 0.30 (0.20-0.44) | 0.29 (0.19-0.42) | 0.26 (0.17-0.41) | <0.001 | 0.73 (0.66-0.80) |
| **Neurodegenerative disease** |  |  |  |  |  |  |  |
| No. of cases/person-years | 17/21,256 | 24/37,196 | 37/59,422 | 39/63,364 | 31/48,925 |  |  |
| Hazard ratio (95% CI) | 1 | 0.70 (0.37-1.30) | 0.63 (0.36-1.12) | 0.57 (0.32-1.03) | 0.55 (0.30-1.00) | 0.070 | 0.89 (0.78-1.01) |
| **Digestive disease** |  |  |  |  |  |  |  |
| No. of cases/person-years | 37/21,256 | 34/37,196 | 25/59,422 | 24/63,364 | 7/48,925 |  |  |
| Hazard ratio (95% CI) | 1 | 0.62 (0.39-0.99) | 0.32 (0.19-0.55) | 0.31 (0.18-0.54) | 0.12 (0.05-0.28) | <0.001 | 0.65 (0.57-0.75) |
| **Other^b^** |  |  |  |  |  |  |  |
| No. of cases/person-years | 56/21,256 | 70/37,196 | 96/59,422 | 81/63,364 | 33/48,925 |  |  |
| Hazard ratio (95% CI) | 1 | 0.82 (0.57-1.16) | 0.77 (0.55-1.09) | 0.66 (0.45-0.95) | 0.35 (0.22-0.56) | <0.001 | 0.83 (0.76-0.90) |
| **Excluding deaths that occurred within the first 4 years of follow-up** | | | | | | | |
|  | **N=1,886** | **N=3,264** | **N=5,186** | **N=5,519** | **N=4,281** |  |  |
| **All cause Mortality** |  |  |  |  |  |  |  |
| No. of cases/person-years | 343/21,065 | 412/36,998 | 470/59,197 | 431/63,197 | 273/48,841 |  |  |
| Hazard ratio (95% CI) | 1 | 0.70 (0.61-0.81) | 0.53 (0.46-0.61) | 0.47 (0.40-0.54) | 0.37 (0.32-0.44) | <0.001 | 0.80 (0.78-0.83) |
| **Cancer** |  |  |  |  |  |  |  |
| No. of cases/person-years | 117/21,065 | 153/36,998 | 185/59,197 | 160/63,197 | 123/48,841 |  |  |
| Hazard ratio (95% CI) | 1 | 0.83 (0.57-1.21) | 0.75 (0.52-1.08) | 0.69 (0.46-1.02) | 0.36 (0.22-0.59) | <0.001 | 0.83 (0.76,0.91) |
| **CVD** |  |  |  |  |  |  |  |
| No. of cases/person-years | 72/21,065 | 90/36,998 | 106/59,197 | 93/63,197 | 55/48,841 |  |  |
| Hazard ratio (95% CI) | 1 | 0.77 (0.56-1.06) | 0.63 (0.46-0.86) | 0.55 (0.40-0.77) | 0.41 (0.28-0.60) | <0.001 | 0.82 (0.76-0.89) |
| **Respiratory disease** |  |  |  |  |  |  |  |
| No. of cases/person-years | 58/21,065 | 54/36,998 | 40/59,197 | 43/63,197 | 29/48,841 |  |  |
| Hazard ratio (95% CI) | 1 | 0.61 (0.42-0.89) | 0.32 (0.21-0.48) | 0.34 (0.23-0.52) | 0.30 (0.19-0.48) | <0.001 | 0.75 (0.68-0.83) |
| **Neurodegenerative disease** |  |  |  |  |  |  |  |
| No. of cases/person-years | 16/21,065 | 23/36,998 | 35/59,197 | 38/63,197 | 30/48,841 |  |  |
| Hazard ratio (95% CI) | 1 | 0.70 (0.37-1.33) | 0.62 (0.34-1.13) | 0.58 (0.32-1.05) | 0.55 (0.29-1.02) | 0.083 | 0.89 (0.78-1.02) |
| **Digestive disease** |  |  |  |  |  |  |  |
| No. of cases/person-years | 31/21,065 | 28/36,998 | 28/59,197 | 21/63,197 | 6/48,841 |  |  |
| Hazard ratio (95% CI) | 1 | 0.58 (0.35-0.97) | 0.30 (0.17-0.54) | 0.32 (0.18-0.57) | 0.12 (0.05-0.28) | <0.001 | 0.66 (0.57-0.76) |
| **Other^b^** |  |  |  |  |  |  |  |
| No. of cases/person-years | 49/21,065 | 64/36,998 | 83/59,197 | 75/63,197 | 30/48,841 |  |  |
| Hazard ratio (95% CI) | 1 | 0.83 (0.57-1.21) | 0.75 (0.52-1.08) | 0.69 (0.46-1.02) | 0.36 (0.22-0.59) | <0.001 | 0.83 (0.76-0.91) |

Abbreviations: CI, confidence interval.

^a^Adjustment for age (years), sex (women or men), ethnicity (White, Black, Asian, Mixed or other), education {higher (college/university degree or other professional qualification), upper secondary (second/final stage of secondary education), lower secondary (first stage of secondary education), vocational (work-related practical qualifications), or other}, Townsend deprivation index, and employment (currently employed or not); adjusting for competing risk of death of other causes.

^b^Mortality from causes other than cancer, CVD, respiratory disease, neurogenerative disease, or digestive disease.

**Table S3.** Hazard ratio (95% CI) of all-cause mortality according to individual modifiable healthy lifestyle factors

| **Modifiable Healthy** **Lifestyle Factors** | **Number of Subjects** | **Number of Cases** | **Person-Years** | **Percentage of**  **Person-Years** | **Hazard Ratio (95% CI)^a^** |
| --- | --- | --- | --- | --- | --- |
| **Smoking** | | | |  |  |
| Never | 11,273 | 937 | 127,663 | 55.4 | 1.00 |
| Previous | 7,222 | 1,029 | 79,864 | 34.7 | 1.46 (1.34-1.60) |
| Current | 2,159 | 481 | 22,891 | 9.9 | 2.48 (2.21-2.79) |
| **Alcohol Consumption (g/day)** | | | |  |  |
| Never | 6,728 | 917 | 74,294 | 32.2 | 1.27 (1.15-1.39) |
| 0-16^b^ | 9,922 | 968 | 112,492 | 48.8 | 1.00 |
| >16^b^ | 4,004 | 562 | 43,631 | 19.0 | 1.36 (1.22-1.51) |
| **Diet** | | | |  |  |
| >=5 recommended components | 11,529 | 1,205 | 129,682 | 56.3 | 1.00 |
| <5 recommended components | 9,125 | 1,242 | 100,736 | 43.7 | 1.22 (1.13-1.33) |
| **Physical activity^c^** | | | |  |  |
| Regular | 10,103 | 978 | 113,631 | 49.3 | 1.00 |
| Irregular | 10,551 | 1,469 | 116,787 | 50.7 | 1.33 (1.23-1.45) |
| **Sleep duration, h/day** | | | |  |  |
| Short, <=6 | 5,903 | 694 | 65,983 | 28.6 | 1.00 (0.91-1.09) |
| Medium, 7-8 | 12,314 | 1,307 | 138,003 | 59.9 | 1.00 |
| Long, >=9 | 2,437 | 446 | 26,431 | 11.5 | 1.39 (1.25-1.55) |
| **Television watching time, h/day** | | | |  |  |
| Never | 619 | 72 | 6,863 | 3.0 | 1.09 (0.86-1.38) |
| Short, 0.5-4 | 11,708 | 1,089 | 131,954 | 57.3 | 1.00 |
| Long, >=4 | 8,327 | 1,286 | 91,600 | 39.8 | 1.17 (1.07-1.28) |
| **Social Connection** | | | |  |  |
| Active | 9,543 | 949 | 107,355 | 46.6 | 1.00 |
| Moderately active | 8,473 | 1,045 | 94,344 | 40.9 | 1.14 (1.05-1.25) |
| Isolated | 2,638 | 453 | 28,718 | 12.5 | 1.34 (1.19-1.51) |

Abbreviations: CI, confidence interval.

^a^Adjustment for age (years), sex (women or men), ethnicity (White, Black, Asian, Mixed or other), education {higher (college/university degree or other professional qualification), upper secondary (second/final stage of secondary education), lower secondary (first stage of secondary education), vocational (work-related practical qualifications), or other}, Townsend deprivation index, and employment (currently employed or not). Lifestyle factors were mutually adjusted for analyses on the association of each individual lifestyle factor with all-cause mortality risk.

^b^Refers to participants who reported drinking frequency $1–3 times/month.

^c^Regular physical activity was defined as >=150 min/week of moderate activity, or >=75 min/week of vigorous activity, or an equivalent combination.

**Table S4.** Hazard ratio (95% CI) of all-cause mortality according to individual modifiable healthy lifestyle factors in male (binary variable)

| **Modifiable Healthy Lifestyle Factors^a^** | **Number of Subjects** | **Number of Cases** | **Person-Years** | **Mean FU time** | **Incidence rate per 1000 person year** | **Hazard Ratio (95% CI)^b^** |
| --- | --- | --- | --- | --- | --- | --- |
| **Smoking** |  |  |  |  |  |  |
| No current | 7,087 | 1,105 | 77531.82 | 10.94 | 14.25 (13.42-15.12) | 1.00 |
| Current | 1,039 | 284 | 10673.64 | 10.27 | 26.61 (23.60-29.89) | 1.72 (1.49-1.98) |
| **Alcohol consumption** |  |  |  |  |  |  |
| Moderate | 4,001 | 603 | 44293.33 | 11.07 | 13.61 (12.55-14.75) | 1.00 |
| Never or Excessive | 4,125 | 786 | 43912.13 | 10.65 | 17.90 (16.67-19.20) | 1.27 (1.14-1.41) |
| **Diet** |  |  |  |  |  |  |
| >=5 recommended components | 3,731 | 578 | 40835.03 | 10.94 | 14.15 (13.02-15.36) | 1.00 |
| <5 recommended components | 4,395 | 811 | 47370.44 | 10.78 | 17.12 (15.96-18.34) | 1.15 (1.03-1.28) |
| **Physical activity** |  |  |  |  |  |  |
| Regular | 3,899 | 532 | 42877.00 | 11.00 | 12.41 (11.38-13.51) | 1.00 |
| Irregular | 4,227 | 857 | 45328.47 | 10.72 | 18.91 (17.66-20.22) | 1.40 (1.26-1.57) |
| **Sleep** |  |  |  |  |  |  |
| Adequate | 4,839 | 732 | 52881.26 | 10.93 | 13.84 (12.86-14.88) | 1.00 |
| Short or long | 3,287 | 657 | 35324.21 | 10.75 | 18.60 (17.20-20.08) | 1.18 (1.06-1.32) |
| **Television watching time** |  |  |  |  |  |  |
| Short | 4,872 | 659 | 53658.70 | 11.01 | 12.28 (11.36-13.26) | 1.00 |
| Long | 3,254 | 730 | 34546.77 | 10.62 | 21.13 (19.63-22.72) | 1.19 (1.07-1.34) |
| **Social Connection** |  |  |  |  |  |  |
| Appropriate | 6,902 | 1,111 | 75276.08 | 10.91 | 14.76 (13.90-15.65) | 1.00 |
| Isolated | 1,224 | 278 | 12929.38 | 10.56 | 21.50 (19.05-24.18) | 1.19 (1.04-1.37) |

Abbreviations: CI, confidence interval.

^a^Low-risk lifestyle factors: no current smoking, moderate alcohol consumption (must be drinking but no more than 2 drinks/day for men on a relatively regular frequency, no drinking is risk factor), healthy diet (adequate intake of at least one-half of 10 recommended food groups), regular physical activity ($150 min/week of moderate activity or $75 min/week of vigorous activity, or an equivalent combination), adequate sleep duration (7–8 h/day), short television watching time (<4 h/day), and appropriate social connection (not isolated).

^b^Adjustment for age (years), ethnicity (White, Black, Asian, Mixed or other), education {higher (college/university degree or other professional qualification), upper secondary (second/final stage of secondary education), lower secondary (first stage of secondary education), vocational (work-related practical qualifications), or other}, Townsend deprivation index, and employment (currently employed or not). Lifestyle factors were mutually adjusted for analyses on the association of each individual lifestyle factor with all-cause mortality risk.

**Table S5.** Hazard ratio (95% CI) of all-cause mortality according to individual modifiable healthy lifestyle factors in female (binary variable)

| **Modifiable Healthy Lifestyle Factors^a^** | **Number of Subjects** | **Number of Cases** | **Person-Years** | **Mean FU time** | **Incidence rate per 1000 person year** | **Hazard Ratio (95% CI)^b^** |
| --- | --- | --- | --- | --- | --- | --- |
| **Smoking** |  |  |  |  |  |  |
| No current | 11,408 | 861 | 129994.85 | 11.40 | 6.62 (6.19-7.08) | 1.00 |
| Current | 1,120 | 197 | 12217.16 | 10.91 | 16.12 (13.95-18.54) | 2.459 (2.083-2.904) |
| **Alcohol consumption** |  |  |  |  |  |  |
| Moderate | 2,956 | 182 | 32817.35 | 11.10 | 5.55 (4.77-6.41) | 1.00 |
| Never or Excessive | 9,572 | 876 | 109394.66 | 11.43 | 8.01 (7.49-8.56) | 1.31 (1.12-1.54) |
| **Diet** |  |  |  |  |  |  |
| >=5 recommended components | 7,798 | 627 | 88846.48 | 11.39 | 7.06 (6.52-7.63) | 1.00 |
| <5 recommended components | 4,730 | 431 | 53365.53 | 11.28 | 8.08 (7.33-8.88) | 1.05 (0.93-1.19) |
| **Physical activity** |  |  |  |  |  |  |
| Regular | 6,204 | 446 | 70753.74 | 11.40 | 6.30 (5.73-6.92) | 1.00 |
| Irregular | 6,324 | 612 | 71458.27 | 11.30 | 8.56 (7.90-9.27) | 1.29 (1.14-1.46) |
| **Sleep** |  |  |  |  |  |  |
| Adequate | 7,475 | 575 | 85122.15 | 11.39 | 6.75 (6.21-7.33) | 1.00 |
| Short or long | 5,053 | 483 | 57089.86 | 11.30 | 8.46 (7.72-9.25) | 1.10 (0.97-1.24) |
| **Television watching time** |  |  |  |  |  |  |
| Short | 7,455 | 502 | 85158.49 | 11.42 | 5.89 (5.39-6.43) | 1.00 |
| Long | 5,073 | 556 | 57053.52 | 11.25 | 9.75 (8.95-10.59) | 1.21 (1.07-1.38) |
| **Social Connection** |  |  |  |  |  |  |
| Appropriate | 11,114 | 883 | 126423.07 | 11.38 | 6.98 (6.53-7.46) | 1.00 |
| Isolated | 1,414 | 175 | 15788.94 | 11.17 | 11.08 (9.50-12.85) | 1.25 (1.06-1.48) |

Abbreviations: CI, confidence interval.

^a^Low-risk lifestyle factors: no current smoking, moderate alcohol consumption (must be drinking but no more than 1 drink/day for women on a relatively regular frequency, no drinking is risk factor), healthy diet (adequate intake of at least one-half of 10 recommended food groups), regular physical activity ($150 min/week of moderate activity or $75 min/week of vigorous activity, or an equivalent combination), adequate sleep duration (7–8 h/day), short television watching time (<4 h/day), and appropriate social connection (not isolated).

^b^Adjustment for age (years), ethnicity (White, Black, Asian, Mixed or other), education {higher (college/university degree or other professional qualification), upper secondary (second/final stage of secondary education), lower secondary (first stage of secondary education), vocational (work-related practical qualifications), or other}, Townsend deprivation index, and employment (currently employed or not). Lifestyle factors were mutually adjusted for analyses on the association of each individual lifestyle factor with all-cause mortality risk.
